# Supplementary material for: Polymorphism analyses and protein modelling inform on functional specialization of Piwi clade genes in the arboviral vector Aedes albopictus
Source: PLoS Negl Trop Dis. 2019 Dec 2;13(12):e0007919. doi: 10.1371/journal.pntd.0007919 (PMC6907866; doi:10.1371/journal.pntd.0007919)
Supplement: S1 Table — (PDF) [file pntd.0007919.s001.pdf]

S1 Table. List of the core components of the piRNA pathway in *Ae. aegypti* and their orthologous in *Ae. albopictus.*

	Aedes aegypti	*Aedes albopictus*
Gene	Gene ID[1]	Gene ID[2]
Ago3	AAEL007823	AALF025916/ AALF025919[4]
Piwi1/3	AAEL013692	AALF005499[5]
Piwi2	AAEL008098	AALF006708[6]
Piwi4	AAEL007698	AALF006337/ AALF008582[7]
Piwi5	AAEL013233	XM_019686836.1/ XM_019692765.1[8]
Piwi6	AAEL013227	AALF016369
Piwi7	AAEL006287	AALF015479/AALF007445[9]

[1]Gene ID is based on AaegL5

[2]Gene ID is based on AaLoF1 for *Ago3*, *Piwi1/3*, *Piwi2*, *Piwi4*, *Piwi6* and *PIWI7*; on canu_80X_arrow2.2 for Piwi5.

[4]In AaLoF1, two genes, AALF025916 and AALF025919, which differ uniquely in the length of their annotated 5'UTR regions, are identified.

[5]Two genes, AALF005499 and AALF005498, are annotated in AaLoF1; sequencing of exon-intron boundaries and cDNA confirmed the gene structure of AALF005499.

[6]Two genes, AALF006534 and AALF006708, are identified in AaLoF1. The only notable difference between the two genes is a tandem duplication of 98bp in AALF006534, which generates a sixth exons. Sequencing of exon-intron boundaries and cDNAs from different mosquitoes confirmed the gene structure of AAELF006708.

[7]In AaLoF1, two genes, AALF06337 and AALF008582, which differ uniquely in the length of their 3'UTR regions, are annotated.

[8]No sequence orthologous to AAEL013233 was retrieved in AaLoF1, but three sequences were identified in the genome of C6/36 cells. XM_019692797.1.

[9]Two genes, AALF015479 and AALF007445, are identified in AaLoF1, with a >96% sequence similarity, suggesting they are alternative alleles of the same gene.
