# Supplementary material for: Polymorphism analyses and protein modelling inform on functional specialization of Piwi clade genes in the arboviral vector Aedes albopictus
Source: PLoS Negl Trop Dis. 2019 Dec 2;13(12):e0007919. doi: 10.1371/journal.pntd.0007919 (PMC6907866; doi:10.1371/journal.pntd.0007919)
Supplement: S2 Table — (PDF) [file pntd.0007919.s002.pdf]

S2 Table. List of transcript IDs and species abbreviations of the Culicinae species included in the phylogenetic analyses.

Order	Family	Subfamily	Species	Trascript (cDNA) ID	Annotation	Code
	Drosophilidae	Drosophilinae	Drosophila melanogaster	FBpp0079755	Piwi	DrMelPiwi
	Drosophilidae	Drosophilinae	Drosophila melanogaster	FBpp0079754	Aubergine	DrMelAub
	Drosophilidae	Drosophilinae	Drosophila melanogaster	FBpp0289159	Argonaute	DrMelAgo3
	Culicidae	Anophelinae	Anopheles arabiensis	AARA011871-RA	Piwi	AnAraPiwi
	Culicidae	Anophelinae	Anopheles arabiensis	AARA000129-RA	Aubergine	AnAraAub
	Culicidae	Anophelinae	Anopheles arabiensis	AARA009401-RA	Aubergine	AnAraAub
	Culicidae	Anophelinae	Anopheles maculatus	AMAM013572-RA	Aubergine	AnMacAubA
	Culicidae	Anophelinae	Anopheles merus	AMEM008202-RA	Piwi	AnMerusPiwi
	Culicidae	Anophelinae	Anopheles merus	AMEM012277-RA	Aubergine	AnMerusAubA
	Culicidae	Anophelinae	Anopheles merus	AMEM005720-RA	Aubergine	AnMerusAubB
	Culicidae	Anophelinae	Anopheles darlingi	ADAC006051-RA	Piwi	AnAdaPiwiA
	Culicidae	Anophelinae	Anopheles darlingi	ADAC008631-RA	Piwi	AnAdaPiwiB
	Culicidae	Anophelinae	Anopheles christyi	ACHR004324-RA	Piwi	AnChrPiwi
	Culicidae	Anophelinae	Anopheles christyi	ACHR007454-RA	Aubergine	AnChrAubA
	Culicidae	Anophelinae	Anopheles christyi	ACHR009351-RA	Aubergine	AnChrAubB
	Culicidae	Anophelinae	Anopheles minimus	AMIN001881-RA	Piwi	AnMinimPiwi
	Culicidae	Anophelinae	Anopheles minimus	AMIN001620-RA	Aubergine	AnMinimAubA
	Culicidae	Anophelinae	Anopheles minimus	AMIN001621-RA	Aubergine	AnMinimAubB
	Culicidae	Anophelinae	Anopheles minimus	AMIN008377-RA	Aubergine	AnMinimAubC
	Culicidae	Anophelinae	Anopheles albimanus	AALB000197-RA	Piwi	AnAlbPiwi
	Culicidae	Anophelinae	Anopheles albimanus	AALB010570-RA	Aubergine	AnAlbAubA
	Culicidae	Anophelinae	Anopheles gambiae	AGAP009509-RA	Piwi	AnGambiaePiwi
	Culicidae	Anophelinae	Anopheles gambiae	AGAP008862-RA	Aubergine	AnGambiaeAubA
	Culicidae	Anophelinae	Anopheles gambiae	AGAP011204-RA	Aubergine	AnGambiaeAubB
	Culicidae	Anophelinae	Anopheles stephensi	ASTE016375-RA	Piwi	AnStePiwi
	Culicidae	Anophelinae	Anopheles stephensi	ASTE009599-RA	Aubergine	AnSteAubA
	Culicidae	Anophelinae	Anopheles stephensi	ASTE008262-RA	Aubergine	AnSteAubB
	Culicidae	Anophelinae	Anophees coluzii	ACOM032408-RA	Piwi	AnColPiwi
	Culicidae	Anophelinae	Anophees coluzii	ACOM040773-RA	Aubergine	AnColAubA

Culicidae	Anophelinae	Anophees coluzii	ACOM038173-RA	Aubergine	AnColAubB
Culicidae	Anophelinae	Anopheles dirus	ADIR006020-RA	Piwi	AnDirusPiwi
Culicidae	Anophelinae	Anopheles dirus	ADIR009721-RA	Aubergine	AnDirusAubA
Culicidae	Anophelinae	Anopheles dirus	ADIR000087-RA	Aubergine	AnDirusAubB
Culicidae	Anophelinae	Anopheles atroparvus	AATE009117-RA	Piwi	AnAtroPiwi
Culicidae	Anophelinae	Anopheles atroparvus	AATE018010-RA	Aubergine	AnAtroAubA
Culicidae	Anophelinae	Anopheles atroparvus	AATE019740-RA	Aubergine	AnAtroAubB
Culicidae	Anophelinae	Anopheles sinensis	ASIS006352-RA	Piwi	AnSinPiwi
Culicidae	Anophelinae	Anopheles sinensis	ASIS009643-RA	Aubergine	AnSinAubA
Culicidae	Anophelinae	Anopheles sinensis	ASIS011042-RA	Aubergine	AnSinAubB
Culicidae	Anophelinae	Anopheles culicifacies	ACUA026491-RA	Piwi	AnCuliPiwi
Culicidae	Anophelinae	Anopheles culicifacies	ACUA001497-RA	Aubergine	AnCuliAubA
Culicidae	Anophelinae	Anopheles culicifacies	ACUA017711-RA	Aubergine	AnCuliAubB
Culicidae	Anophelinae	Anopheles culicifacies	ACUA005616-RA	Aubergine	AnCuliAubC
Culicidae	Anophelinae	Anopheles quadriannulatus	AQUA005807-RA	Piwi	AnQuaPiwi
Culicidae	Anophelinae	Anopheles quadriannulatus	AQUA016119-RA	Aubergine	AnQuaAubA
Culicidae	Anophelinae	Anopheles quadriannulatus	AQUA007857-RA	Aubergine	AnQuaAubB
Culicidae	Anophelinae	Anopheles quadriannulatus	AQUA005063-RA	Aubergine	AnQuaAubC
Culicidae	Anophelinae	Anopheles epiroticus	AEPI004893-RA	Piwi	AnEpiPiwi
Culicidae	Anophelinae	Anopheles epiroticus	AEPI002451-RA	Aubergine	AnEpiAubA
Culicidae	Anophelinae	Anopheles epiroticus	AEPI006652-RA	Aubergine	AnEpiAubB
Culicidae	Anophelinae	Anopheles melas	AMEC001785-RA	Piwil-like	AnMelasPiwiA
Culicidae	Anophelinae	Anopheles melas	AMEC002029-RA	Piwil-like	AnMelasPiwiB
Culicidae	Anophelinae	Anopheles melas	AMEC006632-RA	Aubergine	AnMelasAubA
Culicidae	Anophelinae	Anopheles melas	AMEC021875-RA	Aubergine	AnMelasAubB
Culicidae	Anophelinae	Anopheles funestus	AFUN005296-RA	Piwi	AnFunPiwi
Culicidae	Anophelinae	Anopheles funestus	AFUN000985-RA	Aubergine	AnFunAubA
Culicidae	Anophelinae	Anopheles funestus	AFUN004060-RA	Aubergine	AnFunAubB
Culicidae	Anophelinae	Anopheles farauti	AFAF000295-RA	Piwi	AnFarPiwi
Culicidae	Anophelinae	Anopheles farauti	AFAF010261-RA	Aubergine	AnFarAubA
Culicidae	Anophelinae	Anopheles farauti	AFAF011957-RA	Aubergine	AnFarAubB
Culicidae	Anophelinae	Anopheles albimanus	AALB001790-RA	Aubergine	AnAlbAub

Culicidae	Culicinae	Culex quinquefasciatus	CPIJ002415-RA	Piwi	CuQuiPiwiA
Culicidae	Culicinae	Culex quinquefasciatus	CPIJ002458-RA	Piwi	CuQuiPiwiB
Culicidae	Culicinae	Culex quinquefasciatus	CPIJ002459-RA	Piwi	CuQuiPiwiC
Culicidae	Culicinae	Culex quinquefasciatus	CPIJ005275-RA	Piwi	CuQuiPiwiD
Culicidae	Culicinae	Culex quinquefasciatus	CPIJ012516-RA	Piwi	CuQuiPiwiE
Culicidae	Culicinae	Culex quinquefasciatus	CPIJ017381-RA	Piwi	CuQuiPiwiF
Culicidae	Culicinae	Culex quinquefasciatus	CPIJ010847-RA	Piwi	CuQuiPiwiG
Culicidae	Culicinae	Aedes aegypti	AAEL008098-RA	Piwi2	AegPiwi2
Culicidae	Culicinae	Aedes aegypti	AAEL013692-RA	Piwi3	AegPiwi3
Culicidae	Culicinae	Aedes aegypti	AAEL007698-RA	Piwi4	AegPiwi4
Culicidae	Culicinae	Aedes aegypti	AAEL013233-RA	Piwi5	AegPiwi5
Culicidae	Culicinae	Aedes aegypti	AAEL013227-RB*	Piwi6	AegPiwi6
Culicidae	Culicinae	Aedes aegypti	AAEL006287-RA	Piwi7	AegPiwi7
Culicidae	Culicinae	Aedes aegypti	AAEL007823-RA	Argonaute	AegAgo3

*Three splice variants are annotated, the longest is considered here
