# Supplementary material for: Polymorphism analyses and protein modelling inform on functional specialization of Piwi clade genes in the arboviral vector Aedes albopictus
Source: PLoS Negl Trop Dis. 2019 Dec 2;13(12):e0007919. doi: 10.1371/journal.pntd.0007919 (PMC6907866; doi:10.1371/journal.pntd.0007919)
Supplement: S3 Table — (PDF) [file pntd.0007919.s003.pdf]

S3 Table. Number of non-synonymous mutations found in mosquitoes of the Foshan strain (Foshan) and wild-caught samples from Mexico (Mex) and the island of La Reunion (Reu) divided by type (i.e. missense [M] , frameshift [F], indel [I] and nonsense [N]) and number of sites in which mutations were found in all tested samples.

Gene	Length	Foshan				Mex				Reu				Shared Sites
		M	F	I	N	M	F	I	N	M	F	I	N	
AGO3	2835	6	-	-	-	12	-	-	-	5	-	-	-	2
Piwi1-3	2661	4	1	1	-	15	-	-	-	7	-	-		-
Piwi2	2628	7	1	-	-	16	3	-	-	8	2	-	-	3
Piwi4	2595	20	3	1	-	30	5	-	-	79	9	3	1	10
Piwi5	2841	8	-	4	-	15	-	8	-	7	-	3	-	4
Piwi6	2664	3	-	-	-	11	-	-	-	6	-	-	-	3
Piwi7	1980	15	-	-	-	29	-	-	-	24	-	-	-	10
**Total**		63	5	6	-	128	8	8	-	137	11	6	1	32
