# Supplementary material for: Polymorphism analyses and protein modelling inform on functional specialization of Piwi clade genes in the arboviral vector Aedes albopictus
Source: PLoS Negl Trop Dis. 2019 Dec 2;13(12):e0007919. doi: 10.1371/journal.pntd.0007919 (PMC6907866; doi:10.1371/journal.pntd.0007919)
Supplement: S4 Table — Relative expression values (log10 fold-change) of Piwi genes during development (A) and following viral infection (B) normalized with respect to sugar-fed samples. Samples (2 pools per condition, 15 individuals each) were analysed at 4 days post infection (early infection) and at 14 and 21 days post infection for CHIKV and DENV, respectively (late infection). Each condition was normalized to the corresponding sugar-fed control and compared to the corresponding Blood-fed control. Ovaries and carcasses were analysed independently. * indicates statistically significant difference between infected and non-infected blood-fed samples (ANOVA framework). Relative expression values may mask differences in levels of expression. For instance, the Ct values of Piwi6, Piwi7 and Piwi1/3 in ovaries 4 days post infection with CHIKV were 30, 33.39 and 25.20, respectively. Ovaries of blood-fed samples at the same time point showed Ct values of 30.30, 33.93 and 26.55 for Piwi6, Piwi7 and Piwi1/3. When relative expression was calculated with respect to Ct values of RPL34, fold-changes in gene expression were comparable among the three genes in both conditions, but Ct values clearly indicate that Piwi7 is less expressed than both Piwi1/3 and Piwi6. These considerations were taken into account when describing results. (PDF) [file pntd.0007919.s004.pdf]

S4 Table. Relative expression values (log10 fold-change) of *Piwi* genes during development (A) and following viral infection (B) normalized with respect to sugar-fed samples. Samples (2 pools per condition, 15 individuals each) were analysed at 4 days post infection (early infection) and at 14 and 21 days post infection for CHIKV and DENV, respectively (late infection). Each condition was normalized to the corresponding Sugar-fed control and compared to the corresponding Blood-fed control. Ovaries and carcasses were analysed independently. * indicates statistically significant difference between infected and non-infected blood-fed samples (ANOVA framework). Relative expression values may mask differences in levels of expression. For instance, the Ct values of *Piwi6, Piwi7* and *Piwi1/3* in ovaries 4 days post infection with CHIKV were 30, 33.39 and 25.20, respectively. Ovaries of blood-fed samples at the same time point showed Ct values of 30.30, 33.93 and 26.55 for *Piwi6, Piwi7* and *Piwi1/3*. When relative expression was calculated with respect to Ct values of RPL34, fold-changes in gene expression were comparable among the three genes in both conditions, but Ct values clearly indicate that *Piwi7* is less expressed than both *Piwi1/3* and *Piwi6*. These considerations were taken into account when describing results.

A	EMBRYOS 4-8H	EMBRYOS 8-16H	EMBRYOS16 -24H	LARVAE 1ST INSTAR	LARVAE 4TH INSTAR	PUPAE	ADULT MALES	SUGAR FED FEMALES 48H	SUGAR FED OVARIES 48H	BLOOD FED FEMALES 48H	BLOOD FED OVARIES 48H
**AGO3**	1.59	1.45	1.89	-0.35	-1.32	-1.06	-0.98	0	0.53	0.20	0.77
**PIWI1/3**	0.95	0.21	0.51	-1.59	-2.05	-2.06	-1.34	0	0.81	0.19	1.09
**PIWI2**	0.26	-0.41	0.41	-1.34	-2.17	-2.15	-1.23	0	1.06	-0.12	0.84
**PIWI4**	0.93	1.05	1.62	-1.98	-1.54	-2.98	-1.76	0	-1.04	-0.53	-1.49
**PIWI5**	-0.02	1.25	0.79	-0.80	-1.46	-1.65	-0.93	0	-0.19	-1.21	-1.83
**PIWI6**	2.10	2.80	2.48	0.09	-0.22	-0.52	-0.68	0	0.70	0.20	0.95
**PIWI7**	3.04	2.07	2.28	-1.41	-1.42	-1.36	-1.01	0	-0.72	-0.88	-1.61

B	OVARIES EALY INFECTION				OVARIES LATE INFECTION				
	Sugar-fed	Blood-fed	DENV	CHIKV	Sugar-fed	Blood-fed	DENV	Blood-fed	CHIKV
**AGO3**	0	0.03	-0.02	1.77*	0	-0.08	0.48	-0.09	0.99*
**PIWI1 /3**	0	-0.15	-0.11	1.54*	0	0.06	0.65	-0.27	0.77
**PIWI2**	0	-0.37	-0.16	0.99*	0	-0.22	0.63	-0.54	0.13
**PIWI4**	0	-0.47	-0.59	0.74*	0	0.39	0.68	-0.45	-0.21
**PIWI5**	0	-0.42	-0.06	0.89*	0	0.34	1.09	-0.58	0.32
**PIWI6**	0	-0.08	0.18	1.20*	0	0.26	0.54	-0.2	-0.02
**PIWI7**	0	-0.13	-0.32	1.32*	0	0.48	1.26	-0.19	0

B	CARCASSES EALY INFECTION				CARCASSES LATE INFECTION				
	Sugar-fed	Blood-fed	DENV	CHIKV	Sugar-fed	Blood-fed	DENV	Blood-fed	CHIKV
**AGO3**	0	0.56	2.06*	-0.83	0	0.79	0.60	1.11	1.69
**PIWI1 /3**	0	0.72	2.36*	-1.08*	0	0.90	0.63	0.53	0.43
**PIWI2**	0	0.32	2.26*	-0.54	0	0.9	0.8	0.92	1.44
**PIWI4**	0	0.44	1.49	-1.19*	0	0.65	0.35	0.95	1.69
**PIWI5**	0	0.38	1.89	-0.85	0	0.6	1	0.24	1.85*
**PIWI6**	0	0.91	2.31*	-0.85*	0	1.25	0.46	0.85	1.47
**PIWI7**	0	0.7	1.57*	-0.84*	0	0.94	0.55	0.85	1.04
