# Supplementary material for: Polymorphism analyses and protein modelling inform on functional specialization of Piwi clade genes in the arboviral vector Aedes albopictus
Source: PLoS Negl Trop Dis. 2019 Dec 2;13(12):e0007919. doi: 10.1371/journal.pntd.0007919 (PMC6907866; doi:10.1371/journal.pntd.0007919)
Supplement: S5 Table — (PDF) [file pntd.0007919.s005.pdf]

S5 Table. List of primers used in this study for A) validation of exon-intron boundaries; B) copy number analyses; C) expression profile; D) probes for Northern-Bot

A) Validation of intron-exon boundaries

Primer name	Sequence
AGO3_ATG_F	ATGTCCTCGCGGTTGAATTT
AGO3_ex1_start_R	TTCCCGTTCTGATGACTGGT
AGO3_ex1_junction_F	CTCGCTGAAACGGTCGAAAC
AGO3_ex2_R	TGGGACACGTCGAGATTCAA
AGO3_ex2_F	ATCGTCTGGAAATCTGGCCA
AGO3_ex3_R	GCATCACCTTGAAGTCGCTT
AGO3_ex3_F	AAGCGACTTCAAGGTGATGC
AGO3_ex3_end_R	GGCCCGAGAATACCAGCG
AGO3_ex3_2_F	CAGGTCATCAACGCCAGAAC
AGO3_ex4_R	GCCGTCACCAACACCATC
AGO3_ex4_F	GATGGTGTTGGTGACGGC
AGO3_ex5_R	GCGGTACCAGGAAGTAATCG
AGO3_ex5_F	CATTACCCGTCGCAACCATT
AGO3_STOP_R	TCACAGGTAGAAGAGTTTGTCGTTCAG
Piwi1-3_ATG_F	ATGGCGGACCGACAACCG
Piwi1-3_ex2_R	GTGTGGGCGATCTTGATCTG
Piwi1-3_ex2_F	AGCAGTTGTCCTTGGAGTGT
Piwi1-3_ex2_end_R	GAAATCATTCCGCATCTGATCG
Piwi1-3_ex3_F	CGCGCCATGTCCGAGTAC
Piwi1-3_ex4_R	GCGCTTTTGTAGACCTCGTT
Piwi1-3_ex4_F	CGACACGAACGACAAATCCA
Piwi1-3_STOP_R	CTACAGGAAGTAGAGCTTCTTCT
Piwi2_ATG_F	TGTCCGACCGTCAATCGCAGG
Piwi2_ex2_R	CAACATTGGCTGGTTCGGAT
Piw2_ex2_F	GTTCATCGATTACTACCAGCAGA
Piwi2_ex3_R	ATCCACACAGCACTTCTTCTTG
Piwi2_ex3_F	TGACGAATCAGAAGGCCGAT
Piwi2_STOP_R	CTACAGGAAGTAAAGCTTCTTCTCGAGCA
Piwi4_ATG_F	ATGTCTGATCGTGATCGTGAT
Piwi4_ex2_R	GCATTGTGGAGAGAAATCCA
Piwi4_ex2_F	CGTGAAAGATCCGGCAATGG
Piwi4 ex2_end_R	CTCGTGACAGCAACATAGGC
Piwi4_ex2_end_F	GCCTATGTTGCTGTCACGAG
Piwi4_ex4_R	TTCGAATCGCATACCACGTG
Piwi4_ex4_F	GGATTGGACGGCTCATTTCC
Piwi4_ex4_2_R	AGCTTTGACGGTGTTGATCG
Piwi4 ex4_2_F	CGATCAACACCGTCAAAGCT
Piwi4 ex5_R	AGTACAGCTTCTTTTCGAGCA
Piwi4 ex5_F	CGATTTCTACCTTGTGTCGCA
Piwi4_STOP_R	TTACAAGAAGTACAGCTTCTTTTCG
Piwi5_5UTR_F	TTTTATGTTACTGTGCGAGCGA

Piwi5_cds_1R_R	TCACCACTTCCAGTAGCACC
Piwi5_cds_1F_F	CGGTGGTGGTGGCTATGG
Piwi5_cds_2R_R	CGGATCGCAACTTATGTCTGG
Piwi5_cds_2F_F	CAGGGGCTGTACAACGTACA
Piwi5_cds_3R_R	AGTTGTCCCTCCAGTTTCCG
Piwi5_cds_3F_F	GGTACCCAGCTGTTCACCA
Piwi5_cds_4R_R	CGAACAAGCTCCGGAACAAG
Piwi5_cds_5F_F	TATGCTGGTGTCTCGCGC
Piwi5_cds_5R_R	CCGGCCGTCACTTTGTAATT
Piwi5_cds_6F_F	GCTGGATCGACGTCTGGTAG
Piwi5_cds_6R_R	AGCCTTATCGTTGCTCACCA
Piwi5_cds_7F_F	CCAACCGTGTAACCATCCAG
Piwi5_cds_7R_R	GCCATACATTGACGCAACCA
Piwi5_cds_8F_F	GCAAACTGGGCGGTATACC
Piwi5_cds_8R_R	CGTACTGCAGTTGACCTTCG
Piwi5_cds_9F_F	AGCTTTGCATTCATATCGGAGT
Piwi5_cds_9R_R	GAATGTTGTAGCTGGTCGGC
Piwi5_cds_10F_F	GACGATATCATCACGCTGCC
Piwi5_cds_10R_R	TACCAAACGCGGAAGGAATC
Piwi6_ATG_F	ATGGCCGATAATCCCCAGGAG
Piwi6_ex3_R	ACCAAACACATGATCAGCTGG
Piwi6_ex2_F	CTTCGACCCGGAAGCTAAAATC
Piwi6_STOP_R	CTACAAAAAGTAAAGTTTCTTCTCCAACCAC
Piwi7_ex1_F	CGTGCTCGCGGTAATGTAAA
Piwi7_ex1_R	TGTACCCTCCAATCGTAGCC
Piwi7_ex1_middle_F	ACGAAACGGCCCTGATGATA
Piwi7_ex1_end_R	GGAAGACCTGCACGAATGTC
Piwi7_ex1_end_F	AACCGCACCTATACCGTCAA
Piwi7_ex2_R	TAGTTGGGTTGCTCGGGATG
Piwi7_ex2_F	TGAACGCCTCGAAGTTTTCA
Piwi7_ex3_R	TGCAGTTGACTTGTATGGCC
Piwi7_ex3_F	GTGCTTGGTTACGAACGACA
Piwi7_ex3_mid_R	CCGTTGGAGTGGTGGTTAAC
Piwi7_ex3_mid_F	GTCCAAATCGTACGGAGCAC
Piwi7-STOP_R	TTACAAGTAATGCAACTTCTTCTC

B) Copy Number

**Primer name**	**Sequence**
AGO3_CN_F	CACCATTACCCGTCGCAAC
AGO3_CN_R	CGGGGACTCGTACACTTCC
Piwi1-3_CN_F	AGGTCAAGATTCCCCTCAGC
Piwi1-3_CN_R	AAGAACTTCGGAGCCTCTGT

Piwi2_CN_F	ACGATTTCCTTGGCTGCTTG
Piwi2_CN_R	GCCTTCTGATTCGTCACCAC
Piwi4_CN_F	AAAACTGCTCTTTTCGCCCG
Piwi4_CN_R	ATCAAACAGCGGCGTCATTT
Piwi5_cds_3_F	GGTACCCAGCTGTTCACCA
Piwi5_cds_3_R	AGTTGTCCCTCCAGTTTCCG
Piwi6_CN_F	TTGAACCCGGACAAGCGTAT
Piwi6_CN_R	TGGAAGAACACCGTCTCCG
Piwi7_CN_F	AGCGCCGAAGTTTTCAGTTT
Piwi7_CN_R	TAGTTGGGTTGCTCGGGATG
aegSCF20(KDR)_F	GACAATGTGGATCGCTTCCC
aegSCR21(KDR)_R	GCAATCTGGCTTGTTAACTTG

C) Expression

Primer name	Sequence
AGO3_qPCR_F	CTCGCTGAAACGGTCGAAAC
AGO3_qPCR_R	CTTTCGAGTCCACCAACGGA
Piwi1-3_qPCR_F	AATTTGATCCTCCGTCGTGC
Piwi1-3_qPCR_R	CGATCTCACAGCAAACGAGG
Piwi2_qPCR_F	GCGAGCAGTTGTCTTTGGAA
Piwi2_qPCR_R	ACAAGTTTCGTCCAACCAGC
Piwi4_qPCR_F	CAACACTCAATCCTCTACACTCTA
Piwi4_qPCR_R	TCACGATCAGACATTATGCTAGAA
Piwi5_cds_3_F	GGTACCCAGCTGTTCACCA
Piwi5_cds_3_R	AGTTGTCCCTCCAGTTTCCG
Piwi6_qPCR_F	TTGAACCCGGACAAGCGTAT
Piwi6_qPCR_R	TGGAAGAACACCGTCTCCG
Piwi7_qPCR_F	GAACAATCATCATCTGCAGTACC
Piwi7_qPCR_R	TTCGTCTGGAGCATTATCTGTC

D) Northern Blot probe

Primer name	Sequence

Piwi5_probe_F	GGTACCCAGCTGTTCACCA
Piwi5_probe_R	CGAACAAGCTCCGGAACAAG
