# Supplementary material for: Polymorphism analyses and protein modelling inform on functional specialization of Piwi clade genes in the arboviral vector Aedes albopictus
Source: PLoS Negl Trop Dis. 2019 Dec 2;13(12):e0007919. doi: 10.1371/journal.pntd.0007919 (PMC6907866; doi:10.1371/journal.pntd.0007919)
Supplement: S1 Dataset — The sequence of the PAZ, MID and PIWI domains is in bold, underline and bold-italics, respectively. (PDF) [file pntd.0007919.s006.pdf]

S1 dataset. Sequence of the transcripts. The sequence of the **PAZ**, MID and ***PIWI*** domains is in **bold**, underline and ***bold-italicus***, respectively.

\> AGO3

ATGTCCTCGCGGTTGAATTTAGTTCGTGCATTTCTCAGTCAATCGGGATCATCCGGAGATGCCGCCGAGCGAACGAAGGC
TCCGTCTTCCGGTGATGCTCCCAGCGAAGGAGTCGATAGTGGTTTCCAAACGCGCACTCCGGAGACCACACCAACCACGC
AGCGCAAAGGTATTGGGCGTGGTCAGCTGACGTCGGATGCGGGAAGAGCAACCGGAACATCCGGAGCAGACTCGTCAGAG
TCCGACGACAAACAGTCCAGCATTGGATCCGCGTTGCCGTCGTTGAGTGGCCGTGGCCGAGCCCAGTTCATCCAAGCGCT
CATTCGCCAGCCGTTCGAACCGGCTCCTTCGGTGGTTTCGGATGATTCGTCTTCCATGGTGTCGGCCAGAGTGTCGCAAA
TTGCCTGCGGACGGGGACGGTTCATTCAACAGCTGCTGAACACGGCTGCCGATGCCGAGAGTATCGAAACCCAATCGAAC
GGAAAACATGACGAACTGTCCGAAGCAGTGTCACAGGTCACGCTCGCTGAAACGGTCGAAACGGAGGAAAAGGCCCCAGT
CATCAGAACGGGAACCAGCGGAACTCCCGTTCAACTGATGACCAACTATATCCGACTGGCATGCGACCCGGACCGCGGTA
TCTACGAGTACGAAGTACGATTCCATCCGTTGGTGGACTCGAAAGCAATCAGAGCTCGCTACATCGCACAGCACAAGGAC
GTTCTCGGTAATGCCAAAACGTTCGACGGCGTCAAGCTGTTTCTTCCGAAGAAGCTTCCAGAGGCGGAACTGATTCTGCA
ATCGAAAAACCCCGTCGACGGCCACGACGTGACCATCAAGATCATCTACAAGCGCAAGCAGCGAATGAGCGAGAACATCC
AGTTCTACAATATTCTCTTCCAGCGCATCATGAAAGTGCTGAAAATGGTCGAGATGGGTCGGAAGAATTTCGACCCGTCG
GCGCCGAAGCTGATTCCGCAGCATCGTCTGGAAATCTGGCCAGGATACGTTAGCGCCGTCGACGAATACGAGGGAGGCTT
GATGTTGAATCTCGACGTGTCCCATCGGGTGCTGCTCCAGACG**ACGGTGTTGGATCACATCCGTACCCTGGCGAGGGCCA**
ATCCGCAGGATTACAAGAACATGGCCACCAAATCGCTTCTGGGAGCCGTCATATTGACTCGCTACAACAACAAAACGTAC
CGCATCGATGACATCCTGTTCGATCAGAATCCCACCATGACATTCGAAGCCAACGGAAAACCCATTTCGTACGTGCAGTA
CTACAAACAGCAGTACAACATCGACATTCACGATCTGAAGCAGCCGCTGCTGATCAACCGGAAGGAACGTCGAGTGGCCG
GCCAGGACAAACCGTTGGAGATGATCATGTGTTTGATCCCGGAGATTTGCTACCTGACCGGATTGACCGACGAGATGCGA
AGCGACTTCAAGGTGATGCGGGACATTGCGGCGTTTACGAGGGTGTCCCCGAATCAGCGCTTGAACAGCATGCGTCAGTT
TTGTCAAAACGTCAACGAAAATAAGGAAGCTCGAGAAATCCTGGAGGTTTGGGGCCTAACGTTGGACATGAACGCCCTGG
TAATGAAGGGACGTTGCTTCAAGGAGGAACAGGTCAACTTCAGCGGGGTCAATGTCCCCGTTGGAAAAGGAGGTGATTTC
AATCGAGCCGTCACCAGCAACAACGTCTTGCAAGCGGTCAACATTCGCAACTGGCTGTTGGTTCACACGGCGAAGGACAC
CCGGATTGCCAAGTCGTTCATGGATTGCGTCGAGCGCAGCTGTCGTCCAATGGGCATTCAGATCGGGCCGCCAGCTATTG
AAGTCCTGCAAACCGATAAGACCGAGCTGTACGTGCAACTGCTACGAACGAAAATCCGCCAAGAAACGCAAATCGTTGTC
ATAATCTGTCCGACCTCACGAGACGATCGCTACGCTGCCATCAAACGTATTTGCTGCTCGGAGATCCCCGTTCCATCGCA
GGTCATCAACGCCAGAACGCTGAGCAACGAGGCCAAGAACCGAGCCATCGTACAAAAGATCATCCTTCAGATGAACTGCA
AACTCGGAGGAACACTG***TGGAGCATTCGCATTCCGTTCGACAATGTGATGATTGCCGGAATCGATACGTACCACGACCCG***
AAACAGAAGAGCAACTCCGTGTCGGCGTTTGTGGCCTCGCTGAATGGCGACTACACCCGCTGGTATTCTCGGGCTTGCAT
TCAGAGCAAAAAGGAGGAGTTCATCAACGGTCTTTGTGCATCGATGGAGAAGTCCTTGAAGGCATACCAGAAGGCAAACT
GCCAGCTGCCGAAAAAGATCATCATTTTCAGAGATGGCGTTGGTGACGGCCAGCTGCGAATGTGTTCCGAGTATGAAATT
CCTCAACTGCTAGAGTCGTGTAAGCTCGTGGAACCGGACTACATTCCGGAGATCACGTTTATTGTCGTCCAAAAGCGTAT
CAATACCCGAATGTTCCGCATCGATGGAAACAACCTGGAAAATCCGAACCCGGGCACCGTTCTGGACCACACCATTACCC
GTCGCAACCATTTCGATTACTTCCTGGTACCGCAGTCGGTCCGTCAGGGTAGCGTTTCACCAACCCACTACATTGTGGTG
CACAACCAGTCGAACCATTCGCCGGACATTCTGCAACGGTTGAGCTACAAGCTATGCTACCTGTACTACAACTGGCCCGG
AAGTGTACGAGTCCCCGCTTGCTGTCAGTATGCCCACAAAATGGCCTACCTCATCGGACAATCGGTCAAACGGAACCCGG
ACGAGACGCTGAACGACAAACTCTTCTACCTGTGA

\> Piwi1/3

ATGGCGGACCGACAACCGAGGAAAACTCGAGCTCGGGGATATACGGCAACGATCAGCTCGTCAGCCGATGCCCGGGGAGG
CCGAGGACAGGCTCCGGTCCGGGGCGGCGGCGTTGGTGTTTCTGGGCCTCGACCGTCGTTCCAGCATCCGGGAGCGGAAG
GTCGCGCCGTAACATATCATGAAGGTTCAGCCGGTCGTGGAGCAGTTAGTGCTTCGAGCAGCCGTGGAACCGGAAATGGC
AATGGGAACAACGGTGGCGGGGGTGATGGCAACGGGAATGGTGCGGGAGCGGCGGCAAGCCGCGGAGCAATGCGTGGTCG
CCGTGCCGTTGCAGATACCCTCCGGACCCGTGCACTGGATGCCCCGTCGAAACATGGAACCATCGGACAACCGCTCCAGC
TACAAGCGAACTACTTCAAGATATTGAAGCACATCGAGTGGACACTGTATCAGTATCGGGTGGATTTTGCTCCGGCCTGC
GACAGTATCCGGTTGATGCAGGGCCTGATCAGTCAGCACAAGAAGACCTTCGGCGGATATCTGTTTGACGGAACTCAGCT
GTTCATGGTGAACAAACTGCGGAGCGAGCAGTTGTCCTTGGAGTGTCGCCACGAGCGGACGGGAGAAGTGTACCAGATCA
AGATCGCCCACACGGGAACCGTGGACATGACGAATGAAACCGGCATTCAGGTGCTGAATTTGATCCTCCGTCGTGCCATG
GGAGGGCTGAATCTCCAGTTGGTTGGACGGAACTTGTTCGATGCGGCGGCCAAGATCACCATCCGTGACTATCATATTGA
ACTGTGGCCGGGATACGTCACCAGCATCCGCCAGCACGAGCAGGACATCCTCGTTTGCTGTGAGATCGCCCACAAAACCA
TGCGGATGCAA**ACCTGTTACGACGTTCTGCGGGATTGTCGGAACAATGACCGCAATTACAAGGATGCCTTCACTAAGACC**
GTCCTGGGTGTGGTCGTACTGACCGGTTACAACAACAAAACCTACACCATTCACGACGTCTCGTACGACACGACTCCGGC
CAGCACGTTCGAGACCAGAAACGGAAAAATCTCGTTCGTTGAGTACTACAAGCAGAAGTACAACATCCAGATCCGGGATC
CGAACCAGCCGATGTTGCTGTCCCGAGCCAAGAAGCGCGATCTGCGGGCCGGTGGTAACGAGCTGATGGCCCTGGTTCCG
GAACTGTGCCAGATGACGGGCCTTACCGATCAGATGCGGAATGATTTCCGCATGATGCGCGCCATGTCCGAGTACACCCG
GCTCAACCCGGACCGACGCATCGAACGACTGGAAACGTTCAACCGTCGTCTTCAGACCACCGCGGCGAGCATGGAAGTGT
TCCAGGTATGGCAAATGGAGCTGGACAAGCGTCTGGTCGAACTCCCCGGTCGATTGCTGCCCCAAGAAATGATCCAATTT
ACACCGACGGAGAAAGGTGTCCCTGCAGGCGAGAATGCCGATTGGACCCAGCACTTTCGCAACAATCCGATGTTTTCGAC
CATTAAGTTGAACCGCTGGTTCATGATCGTACCGAACAGGGCGCAGCGAGAAGCTAACGATTTCCTTGGTTGCTTAATTC
AGGCCGCCCGTGGAATGCGGTTCGAAATCCAGCAGTGCGAGATGGTCACCATCCCGGACGACCATCCTGGCACCTACGTC

AGGACGTTGGACAATGTGGTGAACAAAGATCCTCAGCTGATCATGTGCGTGGTGTCGAACCAGAAGGCCGATCGGTACAC
GGCCATCAAGAAGAAGTGCTGTGTGGATCGCGCCGTTTCGACGCAGGTCATTTGCCAGAAAACCATCACTCCGAAGGGCG
GCAACGTTCGAACACTCATGTCCGTGGCGACCAAAGTCGTCATCCAGATGAACTGCAAGCTGGGTGGAGTGCCA***TGGAAG***
GTCAAGATTCCCCTCAGCGGATTGATGACGGTCGGTTTTGACGTGTGCCACGACACGAACGACAAATCCAAATCCTACGG
AGCCATGGTAGCCACGTTTGACCACGAAAATACAGAGGCTCCGAAGTTCTTCTCAGCCGTAAGTCAACACAGACATGGCG
AGGAAATTTGCAATTACCTCCCGCTGAACACGATCAAGGCGCTCAACGAGTACCGCAAGGAGTACGGTGTGCTTCCGAAG
CGTATCCTCTTCTACCGGGACGGCGTCGGCGAGGGTCAGCTGCACTACGTGTACGAACACGAGGTCAAATCGATTATTGG
GAAACTGAACGAGGTCTACAAAAGCGCCGGAGTCGAGCAGGATGCCTTGTTCACCTTCATCATAGTGAACAAACGCATCA
ACACGAGATTCTTCGATCATAAGCAAAATCCACGGCCGGGAACCGTGGTCGACGATGTGGTGACCAATCCGGAACGTACC
GATTTCTACATCGTTTCGCAGTCGGTCAGACAGGGAACGGTGTCCCCCACTGCCTTCAACGTCCTGTACGACACGTCCGG
CCTCAAGATAGACCATCTGCAGATGTTGTCCTACAAGCAGTGCCATCTGTACTACAACTGGTCCGGAACTGTGCGGGTAC
CGGCCGTATGCCAGTACGCCCACAAGCTGGCTTTCCTGGTCGGGCAGTTCATCCATCAGGCACCGAGCAATCTGCTCGAG
AAGAAGCTCTACTTCCTGTAG

> Piwi2
ATGTCCGACCGTCAATCGCAGGGACGCGCCCGAGCTCGCGGCTACACTGCCGTAAACTTGTCCCATGAAGCGCGGGAGGG
CCGTGGTCAGGCTCCCGTCCGGGGAAGCGGCGTCGGTGTCTCAGGGCCTCGTCCAACCTTCCAGCACCCGGGCGCCGAAG
GGCGTGCCATGACCCATCGGGATGCCTCAGCCGGTCGCGGTGCGTCCAGTTCGACCAGTGGAAACGGAAATGGCAATGGT
AATGGGGCAGCCGCTGCGGGACCAAGCCGTGGAGCGATGCGTGGCCGTCGGGGCGTAGCGGATACGCTTCGCACCCGGGC
ACTGGATGCTCCGTCGAAGCAAGGAACCACCGGACAACCGCTGCAGCTCCATGCCAACTACTTCAAGCTGCGAAAGCACA
TCGAGTGGACGCTGTATCAGTACCGGGTGGACTTTGCTCCAACGTGCGACAGTATCCGGCTGATGCAGGGGCTGATCAGT
CAGCACAAGAAGACCTTCGGCGGATATCTGTTTGACGGAACTCAGCTGTTCATGGTGAACAAATTGCGAAGCGAGCAGTT
GTCTTTGGAATGTCGTCACGAGCGGACGGGAGAAGTTTACCAGATCAAAATCGTCCACACGGGAACTGTCGATATGACGA
ATGAAACTGGAATCCAGGTTTTGAATTTGATCCTTCGTCGTGCCATGGGTGGATTGAACCTCCAGCTGGTTGGACGAAAC
TTGTTCGATGCGGCGGCCAAGATCACTATCCGTGACTACCATATCGAACTGTGGCCGGGATACGTTACCAGCATCCGCCA
GCACGAGCAGGACATCCTCGTGTGCTGCGAGATCGCCCACAAAACCATGCGGATGCAA**ACGTGCTATGACATCCTGCGGG**
ATTGTCAGCGGCACGATCGAAGCTACAAGGACGCCTTCACACGGTCCGTCCTGGGCGTGGTCGTACTGACCGGATATAAC
AACAAAACCTACACCATCCACGACGTGTCGTTCGACACGACTCCGTCCAGTACGTTCGAAACGAAAAACGGAAAATCTC
GTTCATCGATTACTACCAGCAGAAGTACAACATTCGGATCCGGGATCCGAACCAGCCAATGTTGCTTTCGCGCGCCAAGA
AGCGCGATCTGCGAGCCGGTGGCAACGAACTGATGGCCCTGGTTCCGGAACTGTGCCAGATGACGGGCCTCACCGAACAG
ATGCGAAGCGATTTTCGAATGATGCGTGCCATGTCCGAGTATACCCGCCTCAACCCGGACCGACGCATCGAACGACTGGA
AACGTTCAACCGTCGCCTTCAGACCACCCCGGCGAGCATTGAAGTGTTCCAGGTGTGGCAGATGGAGTTGGACAAGCGTC
TGGTCGAACTCCCCGGTCGATTGCTGCCCCAGGAAACGATCTACTTCTCGACCACGGCGCCGGGAGTTCCGGCTGGTGAC
AACGCCGATTGGACCCAGCACTTCCGGAACAATCCGATGTTTTCCACCATTCGACTTGACCATTGGTACGTGGTCGTGCC
GAATCGAGCCCAGCGGGAGGCTAACGATTTCCTTGGCTGCTTGATGCAGGCCGCCCGTGGAATGCGGTTCGAAATCCGGC
AGTGCGAATTCGTAACCATCCCGGATGATAATCCCGGCACCTACGTGCGGATGTTGGACAACCTGGTGAACAAGGATCCT
CAGCTGATCATGTGTGTGGTGACGAATCAGAAGGCCGATCGGTACACGGCCATCAAGAAGAAGTGCTGTGTGGATCGCGC
CGTTCCGACGCAGGTCATTTGCCAGAAAACCATCACTCCGAAGGGCGGCAACGTTCGGACACTCATGTCCGTGGCGACCA
AAGTCGTCATCCAGATGAACTGCAAGCTGGGTGGAGTACCA***TGGAAGGTAAAGATCCCCCTCAACGGATTGATGACGGTC***
GGTTTTGACGTTTGCCACGACACGAACGACAAGTCCAAATCCTACGGAGCCATGGTGGCCACGTTTGACCACGATAACCG
AGGCACCCCGAAGTACTTCTCTACCGTGAGCCAACACGGACATGGCGAGGAAATCTGCAACTACCTGCCGCTGAACACGG
TCAAGGCCCTCAACGAGTACCGCAAAGAGTACAACGAACTGCCGAAACGTATCTTCTTCTACCGGGACGGTGTCGGCGAG
GGTCAGCTGCACTACGTGTACGAACACGAGGTCAAATCGATTATTGGGAAACTGAACGAGGTCTACAAAAGCGCCGGAGT
CGAGCAGGATGCCTTGTTCACCTTCATCATAGTGAACAAACGCATCAACACGAGATTCTTCGATCATAAGCAAAATCCAC
GGCCGGGAACCGTGGTCGACGATGTGGTGACCCTTCCGGAACGAACCGATTTCTACATCGTTTCGCAGTCGGTCAGACAG
GGAACGGTGTCCCCCACCGCGTACAACGTCATCCACGACACGTCCGGCCTGAAGGTCGACCACCTGCAGATGTTGTCCTA
CAAGCAGTGCCATCTGTACTACAACTGGTCCGGCACGACGCGGGTACCGGCGGTGTGCCAGTACGCCCACAAGCTGTCCT
TCCTGGTCGGGCAGTTCATCCACCAGGCACCGAGCAATCTGCTCGAGAAGAAGCTTTACTTCCTGTAG

> Piwi4
ATGTCTGATCGTGATCGTGATCGTTACTCTCAAGGGCGCTCCCGCGCTCGTGGTTACGCAGAAGTGGGTTCTCCCCACGA
TGGTCGTGGAAGTCCTGCGTCACGACCAGCCTACAGTTCGCGCGATGAAGGTCGACCGCGGGAATCATCGAGGGACCACC
ACCGGGAATCATCGTCCGGAGGAGGCCGTGAAAGATCCGGCAATGGCAACGGAGGTGAACGCCCGGAACGTGGCGAGCGG
CGTCAACGCAATCGCCGCGGCGTTGGAGATACGCTCCGAACTCGTGCCCTGGATGCTCCCTCGAAGCACGGTACCACGGG
TCAGGCCCTGCAGTTGAACAGTAACTACTTCAAGCTGTTGAAGCACATCGAGTGGACGCTGTATCAGTATCGGTTGGATT
TCTCTCCACAATGCGCCAGTATGCGTCTTATGCAGGGGTTGGTCAACGAGCACAAGAAAATTTTCGGAGGATTCCTGTTT
GACGGAACTCAGCTGTTCATGGTCAACAAGCTACGAAGTGATCAATTGACCTTGCAGTCGAGACACGAGCGTACCGGAGA
TGTTTACCAGCTCCGTATCGTTCACACCGGTTCCGTGGATATGACCAACGAATCCGGTATCCAAGTGTTGAATTTGATTC
TGCGTCGTGCTATGGCGGGTTTGAACCTTCAGCTGGTCGGCCGTAATCTGTTCGATGCGGCTGCTAAGATCGCTATTCGC
GAATACCAGATCGAACTCTGGCCAGGTTACATTACCAGTATTCGTCAGCACGAACAGGACATCTTGGTGTGCTGTGAAAT
TGCGCATAAAACTATGCGAATGCAA**ACCTGCTACGATATTCTGCGTGATTGCCAGAAGCACGATCGTAACTACAAGGATT**
CGTTCAAACGTGCCGTATTGGGTGTCGTTGTATTGACCGGTTACAACAATAAGACCTACACGATTCACGACGTTACCTTC
GAGACCACTCCGGAGAGTACGTTTGACACCAAGGCTGGCAAAACATCGTTCATTGAGTATTACAAACAGAAGTACAACAT

TCGTATTCGTGATCCCCATCAGCCTATGTTGCTGTCACGAGCAAAGAAACGTGACCTGCGCGCCGGAGGCAGTGAGCTGA
TGGCTCTCGTTCCAGAACTATGTCAAATGACGGGATTGACCGATCAAATGAGATCTGATTTCAGAATGATGCGTGCTATG
GCCGATCACACTCGTCTCAACCCGGATCGTCGTATCGAGCGGTTGGAAACCTTCAATCGGCGGTTGCAAACTTCGCCAGA
AAGTATGGAAGTCTTCAAGACCTGGCAGATGGAGCTCGATCGGCGCCTAATCGATCTTCCCGGTCGACTGTTGCCGCAAG
AGATGATCTTCTTCTCTACCACAGCTAATGGCGTACAAGCTGGAGAACAGGCGGATTGGACGGCTCATTTCCGAAACAAT
CCCATGTTCGCGACGGTGCGATTGACCCGATGGTACCTGATCGTGCCGAACCGAGCCTCCCGCGAAGCCAACGATTTCCT
GGGCTGCATGATTCAGGCCGCACGTGGTATGCGATTCGAAATCAGCAACTGCGAAGTGGTTACCATTCCGGACGACAACC
CCGGAACGTACGTCCGGACGTTGGATAACCTACTGAACAAAGATCCGCAGTTGGTGATGTGTGTGGTGACCAATAACAAG
GCCGATCGGTACACGGCCATCAAGAAGAAGTGTTGCGTTGATCGCGCCATTCCCACGCAAGTCATGGTACAGAAAACGAT
CACCCCGAAGGGTGGCAACGTGCGAACGCTCATGTCCGTGGCCACCAAGGTCGTCATCCAGATGAACTGCAAACTGGGAG
GTGTCCCA***TGGAAGGTCAAGATCCCACTGAACGGATTGATGACCATTGGATTCGACGTTTGTCACGATGGAAAGGATAAG***
TCAAAATCCTTTGGAGCCATGGTAGCCACGTTGGATCACGATAACAAGGGCACGCCGAAGTTCTTCTCAACTGTGAGTCA
GCACACACACGGTGAAGAAATTTCCAATTATCTCCCGATCAACACCGTCAAAGCTTTGAACGAATATCGCAAGGAGTTTG
GAGAGCTGCCCAAGCGTATCATCTTCTACCGAGACGGTGTCGGCGAGGGTCAACTGCACTACGTGTACGAACACGAAGTC
AAATCCATCGTCGAGAAGCTGAACCAGATCTACAAATCTGCGGGAATCGACCAGGACGTCCTGTTGACCTTCTTCATCGT
GAACAAACGTATCAATACGCGATTCTTCGACCATCGCCAGAATCCCAGACCCGGTACCGTGGTCGATGATGTCGTCACCC
TCCCAGAAAGAACCGATTTCTACCTTGTGTCGCAATCGGTCCGTCAGGGTACGGTCTCGCCGACGGCGTACAACGTCATC
TACGACACTTCCGGTCTGAAGATCGACCACCTGCAGATGTTGTCGTACAAGCAGTGCCACTTGTACTACAACTGGTCCGG
AACGACCCGTGTCCCGGCGGTATGCCAGTACGCCCACAAACTGTCCTTCCTGGTGGCGCAGTTCCTTCACCAGGCGCCGA
GCAACTTGCTCGAAAAGAAGCTGTACTTCTTGTAA

>Piwi5
ATGGCGGATAGACAACAGGGAAATCGCGGAGGACGCGGTGGTGGTCGAGGCCGCGGAGGCGGTGGCCGAGGTGGTGGAGG
AGGTTTTAATCAGCGAGGTGGAGGAGGTGGTGGCGGCGGTGGTTACAACCAAGGTCGCGGAGGCTCATCATCCGGTCAAG
GCTGGCCGTCCTTGGGGCAACAACAGCCCCAACAACAGTCCCAAGGTTGGAGACAGCAACCGAGAGAACCCCAGCAGCAA
AGCCAGGGTTGGGGACAGCAAAGATCGGCAGATCAGCCGCAGCCGCAGCAACAGCCAGCACCTGTCCATCAATATCAACC
GCAACAGCAACCGCAACCACAGCAACAACCCCAACAACAACAACCTCAACAACAGCCACGGCAGCAAGATGAATCCGCAG
GGGCTGTACAACGTACAGGAGCAGTCCAGCGTCCTCAGCGCGGTGCTACTGGAAGTGGTGAACCACTTGGTCGTGGAGGC
GTACGCAATCAGCGTGTACTGCAGGACATTGTCCGATCGCGGCCACTGGACAGCACCGTTTCGAAACAAGGCAAGACCGG
AACGCCCATCATGCTGAAAACGAATTACTTCCGAGTTCAACGGAGGGACGATGAAGCCATTTTCCAGTATCGCGTTGATT
TTAATCCTTCGGTCGAAAGTAACAAACTTATGCATTCAATCATGCACAATCTGAAGGGTACCATTGGTGGGTACATTTTT
GAAGGTACTCAGCTGTTCACCAGACATAAGTTGCGATCAGATGAAGTTGAAATAACGACCAAAGAGTCTACAACTGGAAA
GGATTACATTGTAAAGCTGCGGAAGGTCGGCGTAATCGATGGAACGAACGAGATGGCGTTCGTAATTTTCAATCTGATCA
ACAGGAAAGCCATGGGTGGTCTGAAGTTGCAGTTGATCGGGCGCAACTATTTTGACCCTGTGGCCAAGGTCGCAATCAGC
CAATACGGTATTGAGTTGTACCCGGGATACGTAACGAGCATTCGCCAACACGAACAGGATGTATTGATGTGCGCTGAGAT
AACTCATCGTGTCATGCGTACGGAC**ACGTGCTACACGATGTTCAAGCAGTGTGCCAACCAGGGCGGAAACTGGAGGGACA**
ACTACAAGCGCATGATCCTGGGAACGGTCGTAATGGCCACCTACGGCAAGAACAATACCTACACGATCGCCGATGTTGAA
TTCAACACAAGTCCAGAGAGTGCATTCGACGCAAATGGTGTGAAGATTACGTTCTTGCAGTATTTTAAGGATCGGTACAA
TATCACCATCCGTGATCCTCGCCAGCCTATGCTGGTGTCTCGCGCCAAGCAGAGAGACATCCGTGCCGGAAAGACGGAAC
TTATTTATCTTGTTCCGGAGCTTGTTCGCGCTACTGGCCTAACCGACGAAATGCGAAAGAACTTCAACTTGATGCGCACG
CTGGCTGATTATACGCGGTTGACGCCGGATAAGCGTATCCAGCGCTTGGAGAATTTCAATCAACGTTTGCAGCAGTCAAA
GGAAAGCTCGGAGATCTTCCAATTCTGGAAAACCGAGCTGGATCGACGTCTGGTAGAGGTTCCAGCCAGAGTTCTTAAAC
CGGAAGAGATCTTCTTCCACCCGCAGCAGGAAAATTACAAAGTGACGGCCGGCGACATGGCAGATTGGCAGATGGCATTC
CGGAACAATCCAATGTACCTCTCCGTTCCACTGGTCAACTGGTACGTTATCGTACCGGCCGGATCGGAGAAGCTCATGGT
TGATTTCATGCAATGTCTGAAGCAGGCATCCAGGGGTATGCGCTTCCAAGTGGAGGACCCCAACCGTGTAACCATCCAGA
ACGACTCCCCGGCGGTGTACGTGGAGAGTCTGAACCAAGTCGTCCAACGTGATCCTCAGTTGATCATGTGCTTGGTGAGC
AACGATAAGGCCGATCGCTATGCTGCCATCAAGAAGAAGAGCTGTGTTGATAGAGCGGTGCCGACGCAAGTCATCAAGGC
CAGAACCATAACGCCCAAGGGTGGAAATGTGCGAACATTGATGTCGGTGGCCACGAAGGTTGCCATCCAGCTGAACTGCA
AACTGGGCGGAATACCC***TGGGTACTGAGGAACCCTCTGTCGTCAGTGATGGTAATCGGGTTCGACGTGTGCCACGATACA***
CGCGACAAGTCCAAGTCGTTCGGAGCGATGGTTGCGTCAATGTATGGCGGCGGTTGCAAACATCCGAAGTACTTTTCGAC
CGTTAATCACCATTCGAGTGGAGAGGAACTCTCGAATTTCATGGCCCAGAACGTAATCAAAGCTTTGCATTCATATCGGA
GTGATTTCGGCAATACATTTCCCCAGCGCATCATCGTCTACCGGGATGGCGTTGGCGAAGGTCAACTACAGTACGTGTAC
CAGCACGAAGTCTCAGCCATGAAGGAGAAATTGAATGTTGCCTTCAAGGACCAGCCGAACTCGTCTCGGTTGACGTTCTG
CGTGGTAAGCAAGCGAATCAATACGCGCCTGTTCCAAGGTGGACAGAACCCGCTGCCTGGAACAATCGTTGACGATATCA
TCACGCTGCCCGAGAGAAACGACTTCTTCCTGGTTTCCCAAAGCGTGCGCCAGGGAACCGTCTCGCCGACCAGCTACAAC
ATTCTTCGGGACGAGTCCGGTTTGAACGCGGATCAGCTTCAGCTGTACACCTACAAGCAGACCCATCTCTACTACAACTG
GTCTGGCACCGTGGGAGTTCCGGCCGTTTGCCAGTATGCCCACAAGTTGGCCTTCCTGGCCGGACAACACCTGCACCAGT
CGCCGACCACTTTGCTCGAGAAGAAGTTATACTATTTGTAA

> Piwi6
ATGGCCGATAATCCCCAGGAGGGCTCCAGTGGCGGCCGGATACGGGCCCGCGGTTTCGTAGGAGGATCCTCATCGCGTGG
CGGCTACAACGGAGGATCTTCACGAGGCGCTTGGCCAGGGTCGGACAATGGAGGCGGATATAGAGGTGATCACCGGCAGA
AGCCTTACGATCGTCCGGAACATTCGCCACCGAGCGTGGCACCAACAGAGGAAGTGTCCGCATTGGAGTCGGAAATTAAG

CAGGAGAAGATGCAGGGCCGAGGTGAGCGTGGTGGCCGAGGAGGCTCCCGAGGTGGTCGTGGTGGACTGCGAGGAAACCG
TTATTTGCCGGAAATTGTGCATACCCGTACGGACACATCGGTTGCCAAGCAAGGAAACACCGGACGACCTATCATGCTTC
AGACCAATTATTTCCGTGTGCTTCGCAATGAGGACGAGCGAATTTTCCATTATCGTGTCGATTTTAATCCCTCGGTGGAA
GCGAACAAGAAAATGCGAGCTCTTATGTTCCAGATCAAGCCACACATTGGAGGGTACGTGTTTGACGGAACGCAGGTCTT
TACGCGAACAAAGTTAGAAAAGGACCCGGCGGAGTTCACAGCTAGGGACAAATTCACCGAAGAAGACCATGTTATTACTT
TGCGACGAGTTGGCGATGTTACCGGTACGAATGAAAAAGCTTTTGTGGTATTCAACCTTATCAACCGTCACGCCATGGCC
GGCCTCAAATTACAACTTATTGGGCGAAGTTTCTTCGACCCGGAAGCTAAAGTCTCAATCAGACAGTACGGAATTGATCT
GTACCCGGGCTATCTCACGAGCATCCGCCAGCACGAGCAGGATGTGCTTATGTGTGCCGAACTGACACACCGGGTTATGC
GCACGGAC**ACGTGCTATTCGCTATTTGAACACTGCATGAATCAACGCGGAAACTTCAAGGACAACTACAAACGCATGGTT**
CTGGGAACTGTGGTAATGTCCACCTATGGCAAGAACAATACTTACACCATCTCGGACGTTGAATTCGACGTCAACCCGGA
GAGCACTTTCGAGACCAAGAAGGGCCCGATCACCTTCATGCAGTACTTCAAGGACAAATACAATATTGTTATTCGGGATC
CGCGGCAACCGATGCTGGTGTCCCGCTCAAAAGCCAGGGACATCCGGGCGGGTTTACCGGAGCTGATCTACCTTATTCCC
GAACTGTCCAGGATCACTGGCATTACCGACGATATGCGACGGGATTTCCATCTCATGCGGGCGATTGCCGACCACACCCG
GTTGAACCCGGACAAGCGTATCCAACGGCTGGAGACGTTCAACCGGCGGATGCAACAGTCCAAGGACAGTTCCGATGTGT
TCAAGTTCTGGAAAACGGAACTGGACCGCAGACTGGTGGAGGTTCCAGCCCGTGTGCTCCCGCCGGAGACGGTGTTCTTC
CACCCGGAGCAGGATCAGTGCAAGGTCCTGGCAGGTGAGATGGCCGAATGGCAAATGGCATTCCGCAACAACCCGATGTA
TCTGACGGTTGCTCTCACCAATTGGTACGTGGTCGTGCCCGGCGGTTCGGAACGGTTGATCGTCGATTTCATGTCCTGCT
TGAAACAAGCCGCTTCACAGATGTATTTCCAAATCGAAGAGCCTCGCCGCATTTCCATTCCCAACGATTCACCCGTCGTC
TACGTGGAGCAGTTGAGCCAGATCGTTCAGCGAGATCCCCAGCTGATCATGTGCTTGGTGACGAACGACAAGGCCGATCG
TTATTCGGCCATTAAGAAGAAATGCTGCGTGGACCGAGCCGTACCGACGCAGGTGCTGAAGACCCGAACGATCACCCCTA
AGGGAGGAAATGTACGTACGCTGATGTCCGTGGCCACCAAAGTAGCGATCCAGATGAACTGCAAGCTGGGTGGAATTCCA
TGGGTCATCAAGAGCCCTCTGGCTTCGGTGATGGTAATCGGATACGATGTGTGCAAGGATTCCAAGGATAGATCGAAAGG
ATACGGAGCGTTGGTGGCCTCCATGTATGGAGGTGGCATCAAACATCCCAAGTACTACTCGACCGTAAACCAGCATGCCC
ACGGAGAGGAGCTGTCCAACTACTTGGCCTTAAATGTTATCAAGGCGATCCGCGCGTATCAGTCGAGCTTTGGAAACATC
CTGCCGCAGCGTATCGTTATCTACCGGGATGGCGTTGGCGATGGGGATTTGGGCTTCGTTTACGAGCACGAAGTCGGTGC
TGTGAAGGAAAAACTGGAAGCCGCTTACAAAGGACAGGATTTCCCGTCGAAGCTGACCTTCTTCGTTGTCAACAAGCGCA
TCAACACGCGACTGTTCCAAGATCGGAGGAACCCAACACCTGGAACCATAGTCGATGACGTCATTACACTACCCGAGAGA
AACGACTTCTACCTGGTTTCCCAGAGCGTCCGGCAGGGTACGGTCTCCCCGACGTCGTACAACATTCTGAGGGACGAATC
CGGCCTCAGTGCCGATCGGTTGCAGTTGTACACCTTCAAGCAGACGCACATGTACTACAACTGGTCCGGTACCGTCGGGG
TGCCGGCCGTTTGTCAGTATGCCCACAAACTGGCCGCCTTGGCGGGACAGTATTTGCATCAAGCCCCGAGCACGTGGTTG
GAGAAGAAACTTTACTTTTTGTAG

>Piwi7
ATGATATTTAACCTGATTAACCGCAAGGCGATGGGTGGGCTGAATTTACAGCTCATCGGAAGAAACTTCTTCGACCCCGC
TGCAAAGTTAACCGTCAGCCAATACGGGATTGAATTATATCCCGGTTATGTGACTAGCATTAGGCAGCATGAGAGGGATG
TTCTGATGTGTGCTGAGCTGACGCATCGAGTCATGCGAACCGAC**ACCTGCTATACTATAATGAAGCAGTGTATGATGCAC**
GGTGGTAACTGGAAGGATAATTTCAAAAGGATGGTACTTGGTTCCGTTGTGATGACTACATATGGGAAGAACCGCACCTA
TACCGTCAACGATGTGGAGTACAACACCACAGCGGAGAGCAATTTCCAGACATCGAGCGGAAACACTACGTTTGTCCAGT
ACTTCAAGGAGCGCTACAACATTATAATCCGTGATCCAAGACAACCGATGTTGGTTTCAAGGTCGAAACCGCGAGACATT
CGTGCAGGTCTTCCCGAGCTTATCTATCTGGTGCCCGAACTAGTCCGTGCCACCGGAATTACCGACGACATGCGGCGGAA
TTTCAATTTAATGCGCACATTAGCGGATCACACTCGTCTGACGCCGGATAGGCGCATTGAACGCCTCGAAGTTTTCAATC
GGCGCCTGCAGGATTCCAAAGAAAGCGCCGAGGTTTTCAGTTTCTGGAAGACGGAACTAGACCGACGACTGGTCGAAGTC
CCGGCGCGAGTGCTTCAACCGGAAACAATATTCTTCCATCCCGAGCAACCCAACTATGCGGTTTCTGCAGGAGAGATGGC
CGAATGGCAGATGGCCTTCCGTAACAACCCCATGTACTACTCGGTGGCCCTTACGCAGTGGTTCGCAGTGGTTCCCAAAG
GATCGGAACGTCTGATCACAGATTTCATGCAATGTCTTCGGCAGGCGGCTCGCGGCATGCGCTTCCAGATTGAGGAACCC
CAGATCGTGGTCATTCCCAACGATTCGCCGGCAGTTTACATCGACAGTCTGAATTCGATCGTTCAGCGGGATCCACAAAT
GATCATGTGCTTGGTTACGAACGACAAGGCCGATCGGTACGCTGCCATCAAGAAGAAGTGTTGCGTCGATCGCGCCGTGG
CAACGCAGGTCATCAAAACTCGTACGATCACACCCAAAGGCGGCAATGTGCGAACGTTGATGTCGGTGGCAACGAAAGTC
GCCATACAAGTCAACTGCAAGTTGGGTGGCATCCCC***TGGATTTTGAAGAACCCCCTGAGCTCCATCATGGTCATCGGGTA***
TGACGTCTGCCATGACACTCGAGACAAGTCCAAATCGTACGGAGCACTTGTAGCGTCGATGTATGGAGCGGGATGTCGAC
ATCCTAAGTATTTCTCCACGGTTAACCACCACTCCAACGGGGAAGAACTTTCCAACTTCATGGCCCAGAACATAATTAAA
GCTCTTCATTCGTATCGTGCTGATTTTGGAAATGCACTACCCGACCGGATCATCGTCTACCGCGACGGAGTCGGAGATGG
ACAGCTCAAGCATGTCTACGAGCATGAAGTAAACTCGATCAAAGACAATCTTCTTCTAGCCTGTAAGGCACAGCCAAACT
CTCCAAAATTAACTTTCTTCGTGGTGAACAAACGCATCAACACCAGGCTGTTCCACCAGAAGCGGAATCCGGTGCCGGGA
ACGATCGTGGATGACGTCATCACCCTACCAGAGAGGAACGATTTCTACCTCGTTTCGCAAAGCGTCCGGCAAGGTACGGT
CTCGCCCACCAGCTACAATATTCTCAAGGACGAATCCGGGTTGAGTGCCGACAAACTGCAGCTGTACACATTCAAGCAGA
CGCACATGTACTACAACTGGTCCGGTACGGTGGGGGTACCAGCGGTTTGCCAGTATGCACACAAGTTGGCAGCACTGGCC
GGACAGTATTTGCATCAAGCTCCCAACAATTTGTTGGAGAAGAAGTTGTATTACTTGTAA
