# Supplementary figures and images for: Polymorphism analyses and protein modelling inform on functional specialization of Piwi clade genes in the arboviral vector Aedes albopictus
Source: PLoS Negl Trop Dis. 2019 Dec 2;13(12):e0007919. doi: 10.1371/journal.pntd.0007919 (PMC6907866; doi:10.1371/journal.pntd.0007919)

S1 Fig

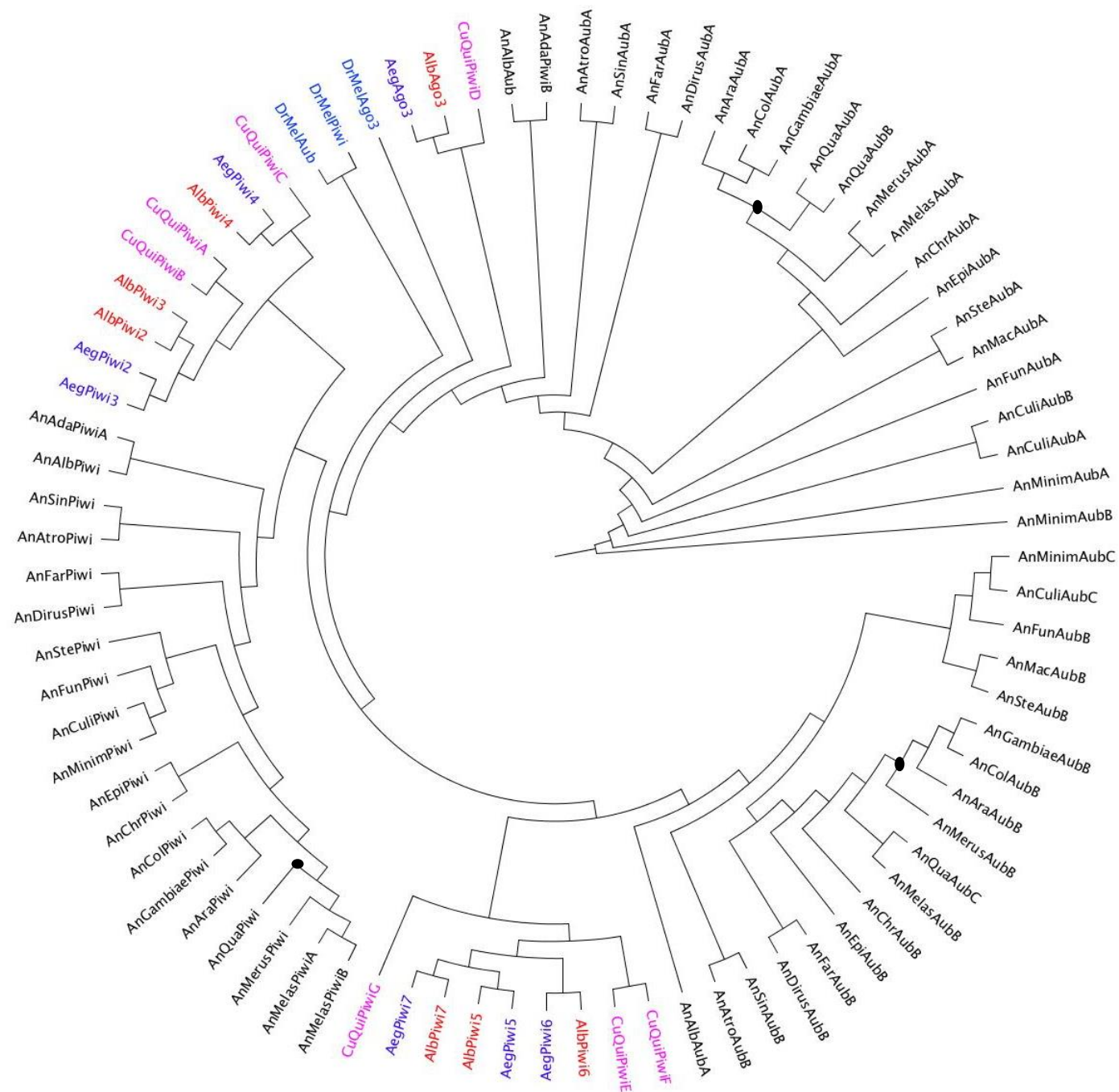

Supplement: S1 Fig — Transcript IDs and species abbreviations are as listed in S2 Table. AlbPiwi3 is the same as Piwi1/3 in the text. Piwi gene transcripts from Ae. albopictus are in red, from Ae. aegypti in purple, from Culex quinquefasciatus in pink. Transcripts from D. melanogaster Ago3, Piwi and Aubergine genes are included for reference and shown in blue. All nodes were supported by bootstrap values higher than 50% with the exception of the three nodes with a black dot. (PDF) [file pntd.0007919.s007.pdf]

S2 Fig

A. Piwi4 CDS

● Missense mutations

● Frameshit mutations

● Indels

● Stop

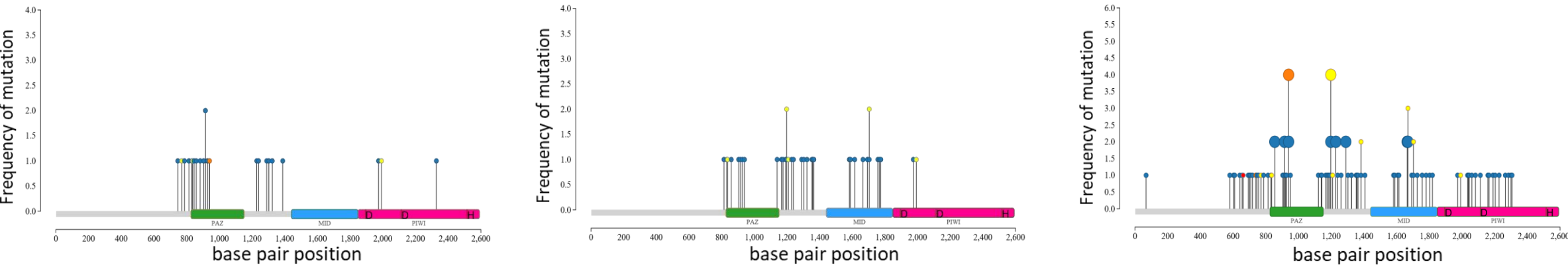

B. Piwi5 CDS

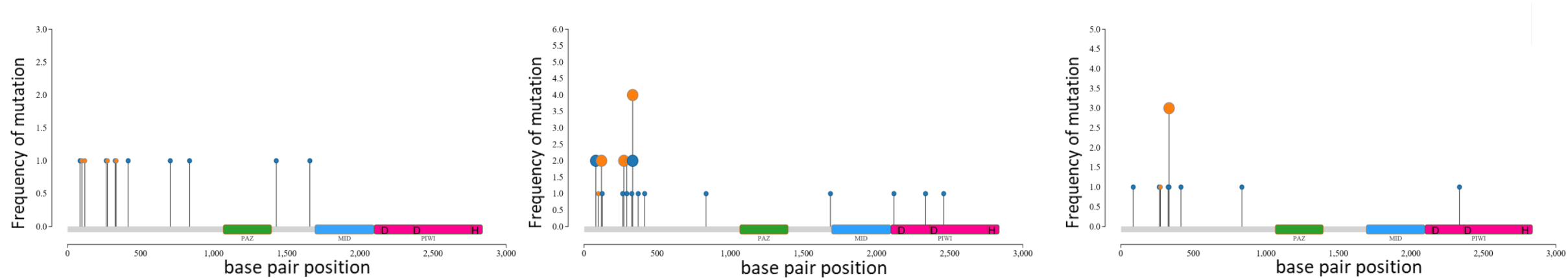

Foshan

Mex

Reu

Supplement: S2 Fig — Lollipop plots representing position, amount and type of mutation along the coding sequences of Piwi4 and Piwi5 in mosquitoes of the Foshan strain, from la Reunion Island (Reu) and Mexico (Mex) as inferred by Freebayes and SnpEFF analyses. Only missense (blue), nonsense (red) and indels (orange) and frameshift (yellow) are shown. The PAZ, MID and PIWI domains are shown in green, blue and magenta, respectively. DDH residues positions are highlighted in the PIWI domain. (PDF) [file pntd.0007919.s008.pdf]

# S3 fig

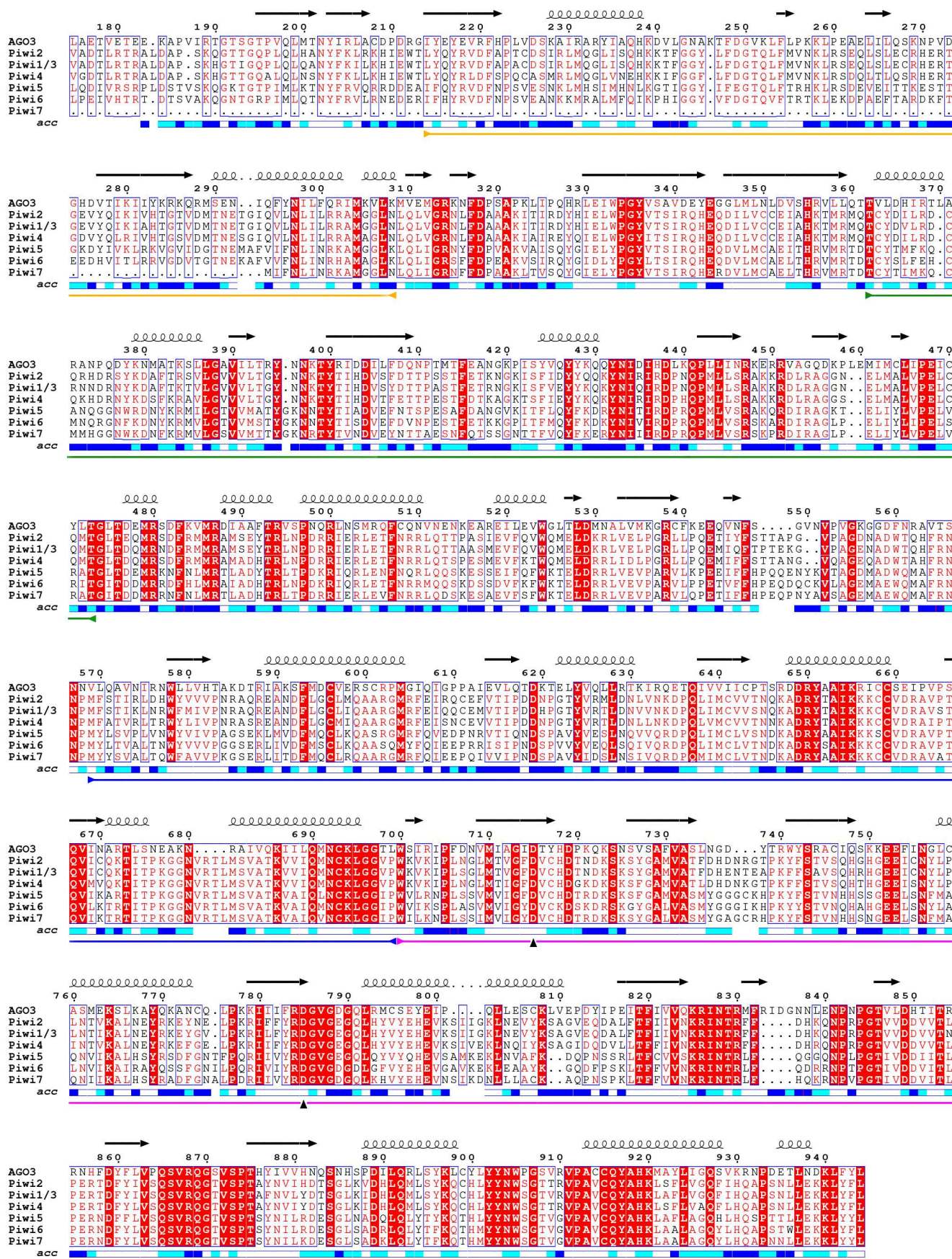

Supplement: S3 Fig — Domain boundaries inferred from structural predictions are highlighted by coloured lines using the same colour coding as in Fig 4 (Orange: N-terminus; Green: PAZ; Blue: MID; Magenta: PIWI). Conserved DDH residues found in PIWI are indicated by a black triangle (▲). The “acc” line indicates relative solvent accessibility, ranging from blue (accessible) to white (buried). The sequence alignment was generated using EBI muscle [88] and depicted using ESPRIPT3 [89]. (PDF) [file pntd.0007919.s009.pdf]

S4 fig

## Tapachula, Mexico

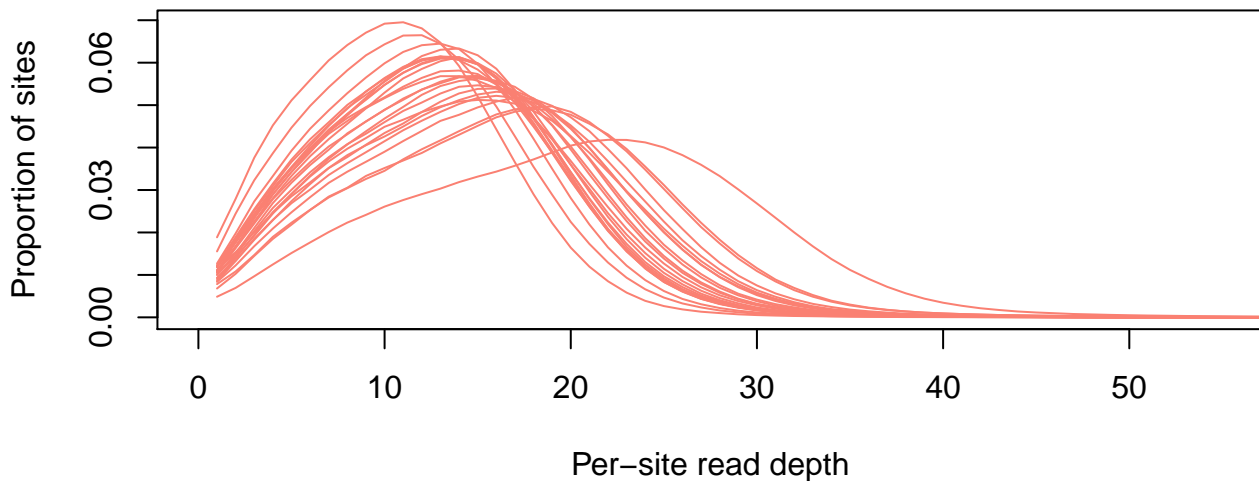

## Le Tampon, Reunion

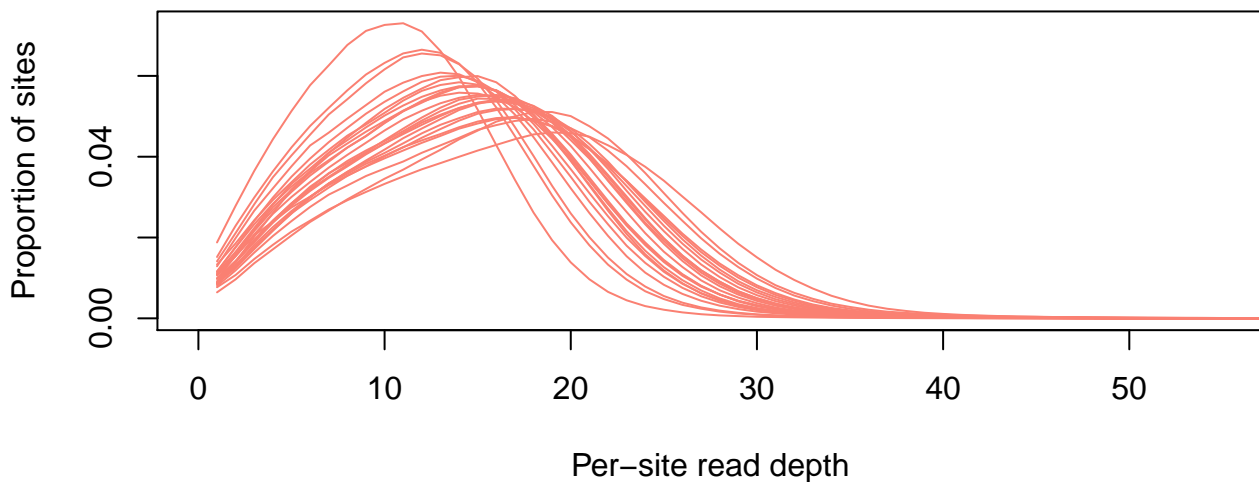

Supplement: S4 Fig — (PDF) [file pntd.0007919.s010.pdf]

S5 fig

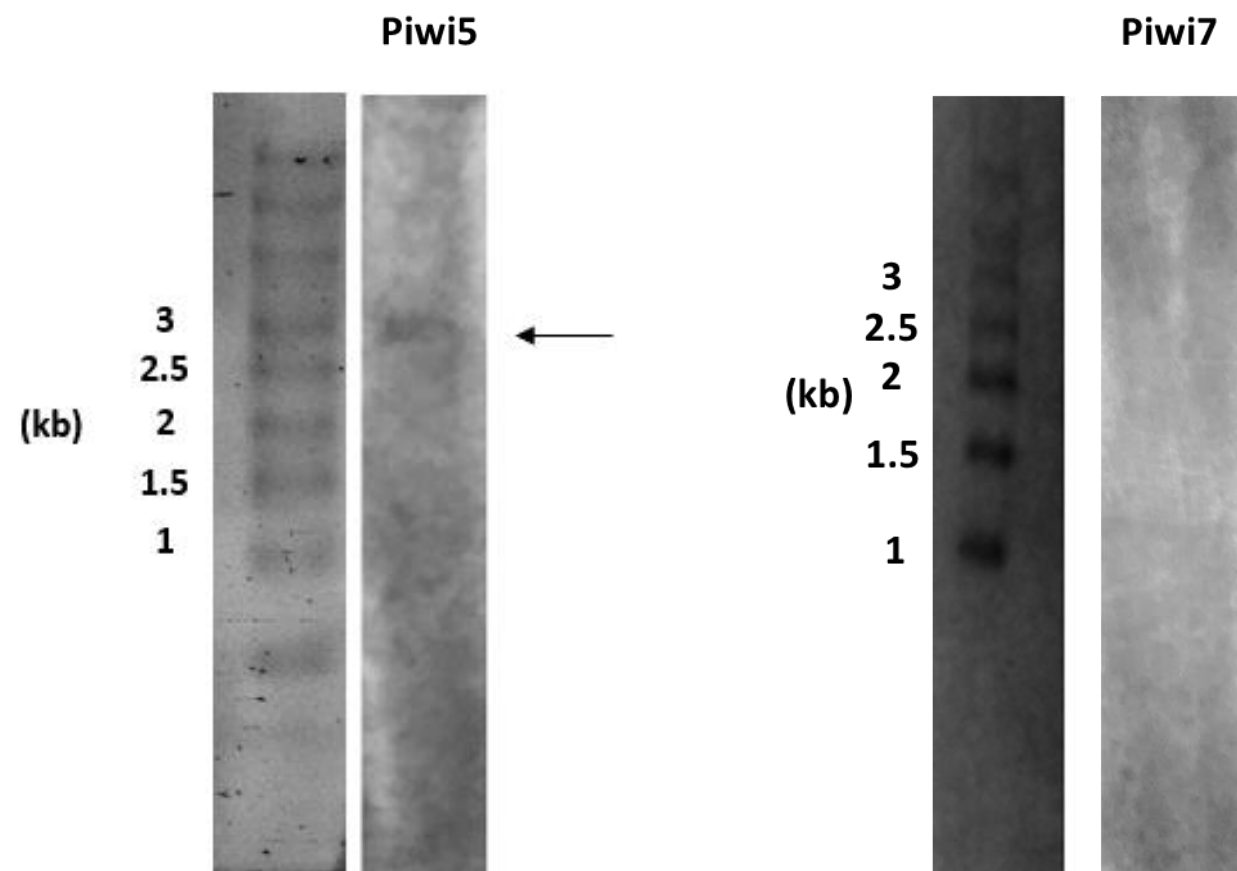

Supplement: S5 Fig — (PDF) [file pntd.0007919.s011.pdf]
